# Supplementary material for: Insights into Two Novel Orthopalladated Chromophores with Antimicrobial Activity against Escherichia coli
Source: Molecules. 2022 Sep 16;27(18):6060. doi: 10.3390/molecules27186060 (PMC9504776; doi:10.3390/molecules27186060)
Supplement: Supplementary file 1 [file molecules-27-06060-s001.zip › molecules-1857961-supplementary.pdf]

Insights into two novel orthopalladated chromophores with antimicrobial activity against *Escherichia coli*

Rosita Diana[1], Francesco Silvio Gentile[2], Antonio Carella[3], Luigi Di Costanzo[1], Barbara Panunzi[1*]

[1] Department of Agricultural Sciences, University of Naples Federico II, Portici, NA, Italy
[2] Department of Pharmacy, University of Salerno, Fisciano, SA, Italy
[3] Department of Chemical Sciences, University of Naples Federico II, NA, Italy

Supplementary Material

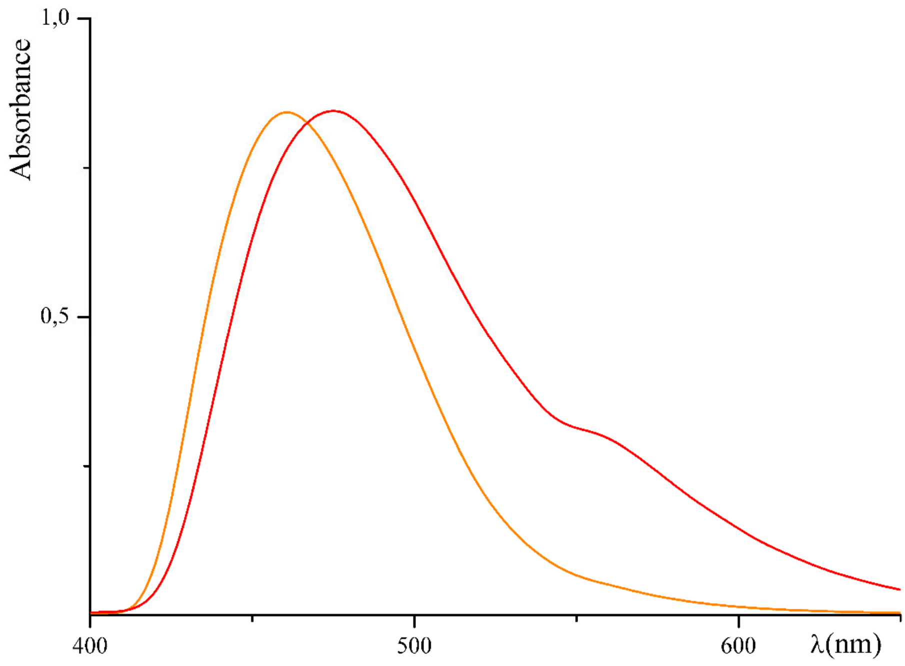

Figure S1. Absorbance spectra of Pd1 (red curve) and Pd2 (yellow curve) in the solid phase.

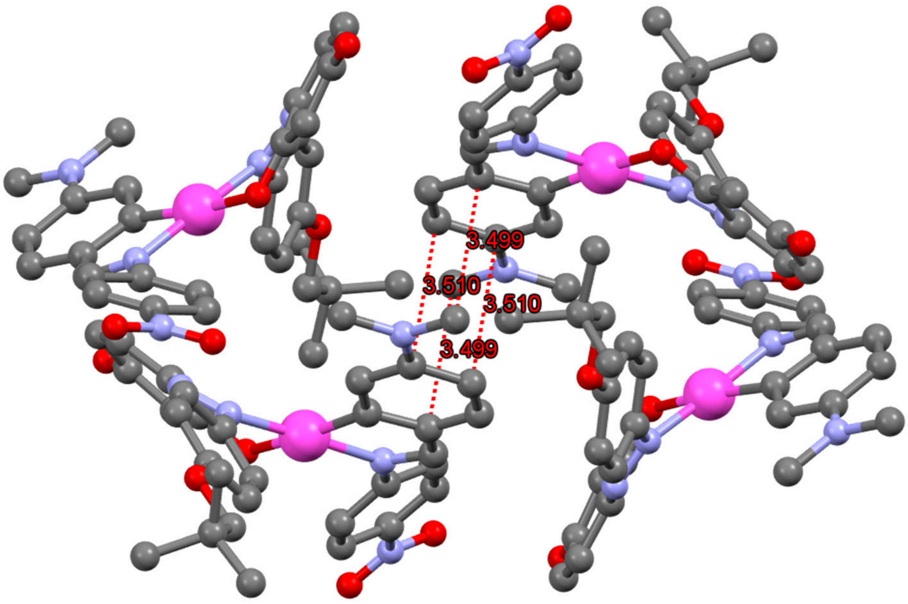

Figure S2. A selection of symmetric short distances $-CH_3$--π and π--π displaced stacking interactions

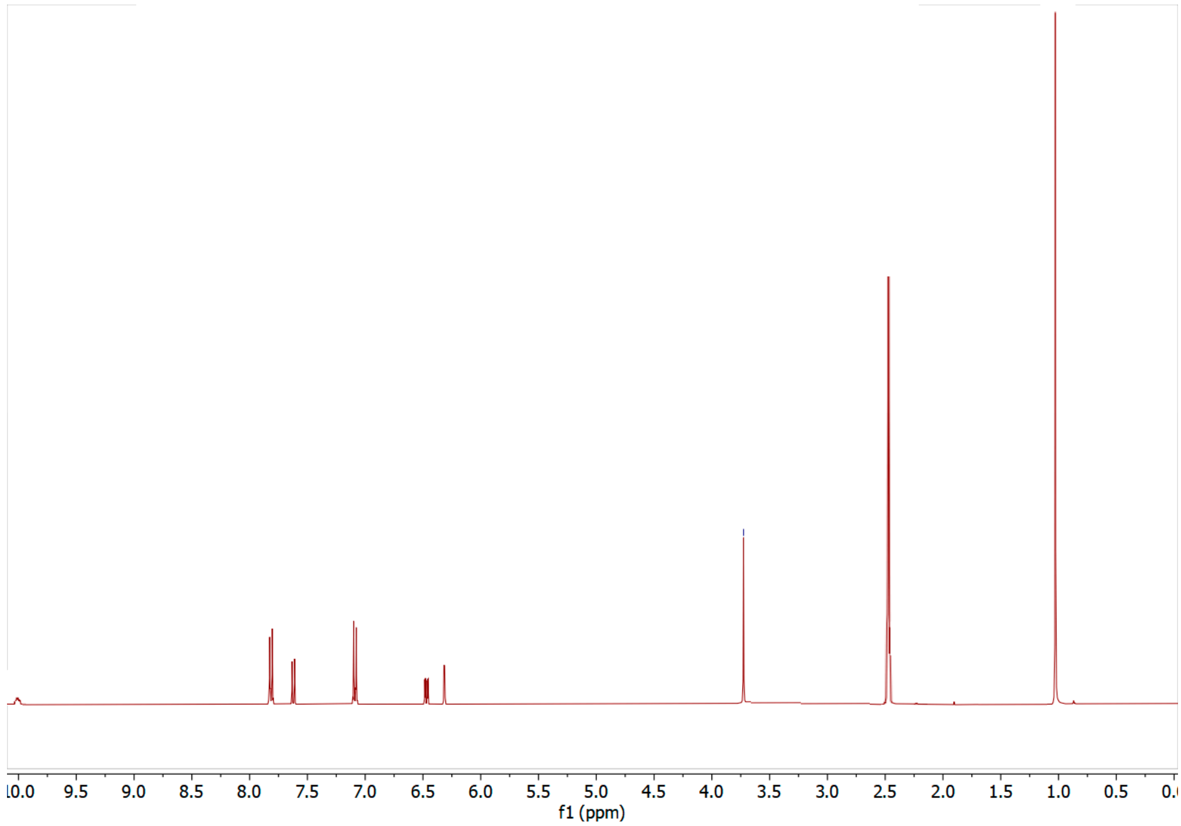

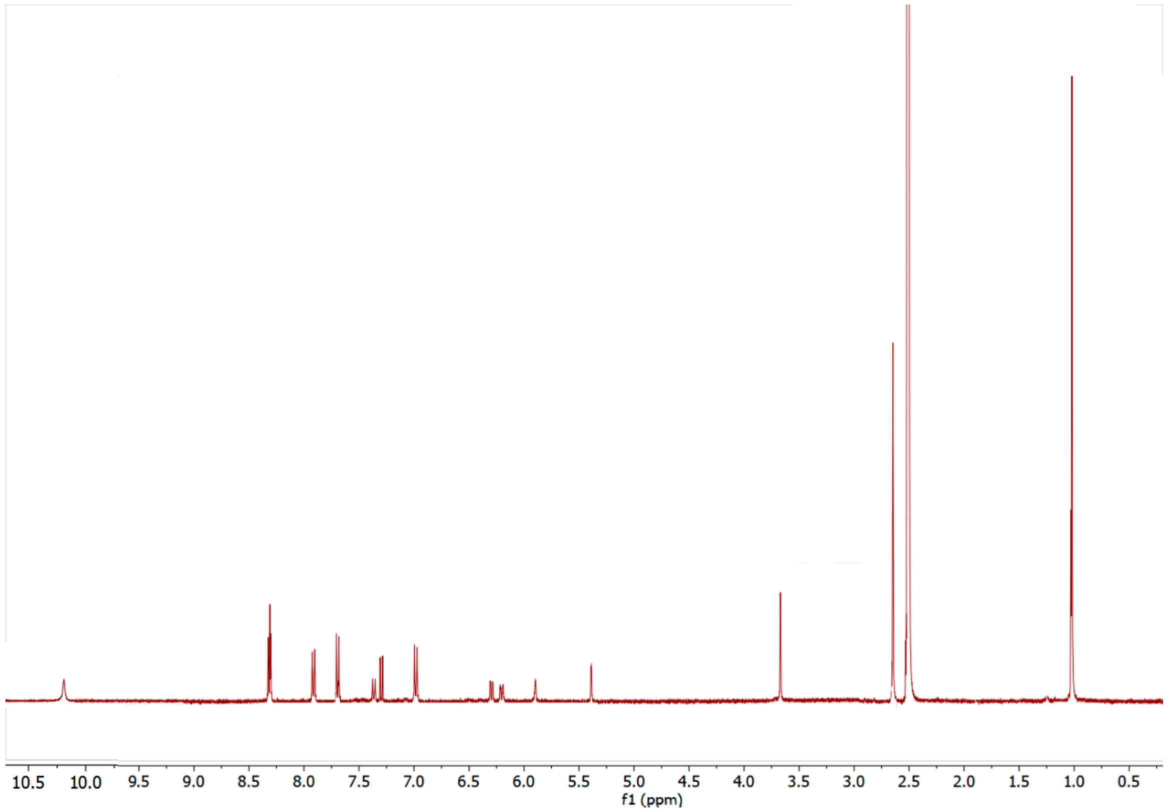

Figure S3. ^{1}H-NMR (400 MHz, DMSO-d6, 25 °C, ppm) spectra of L1 (above) and Pd1(below)

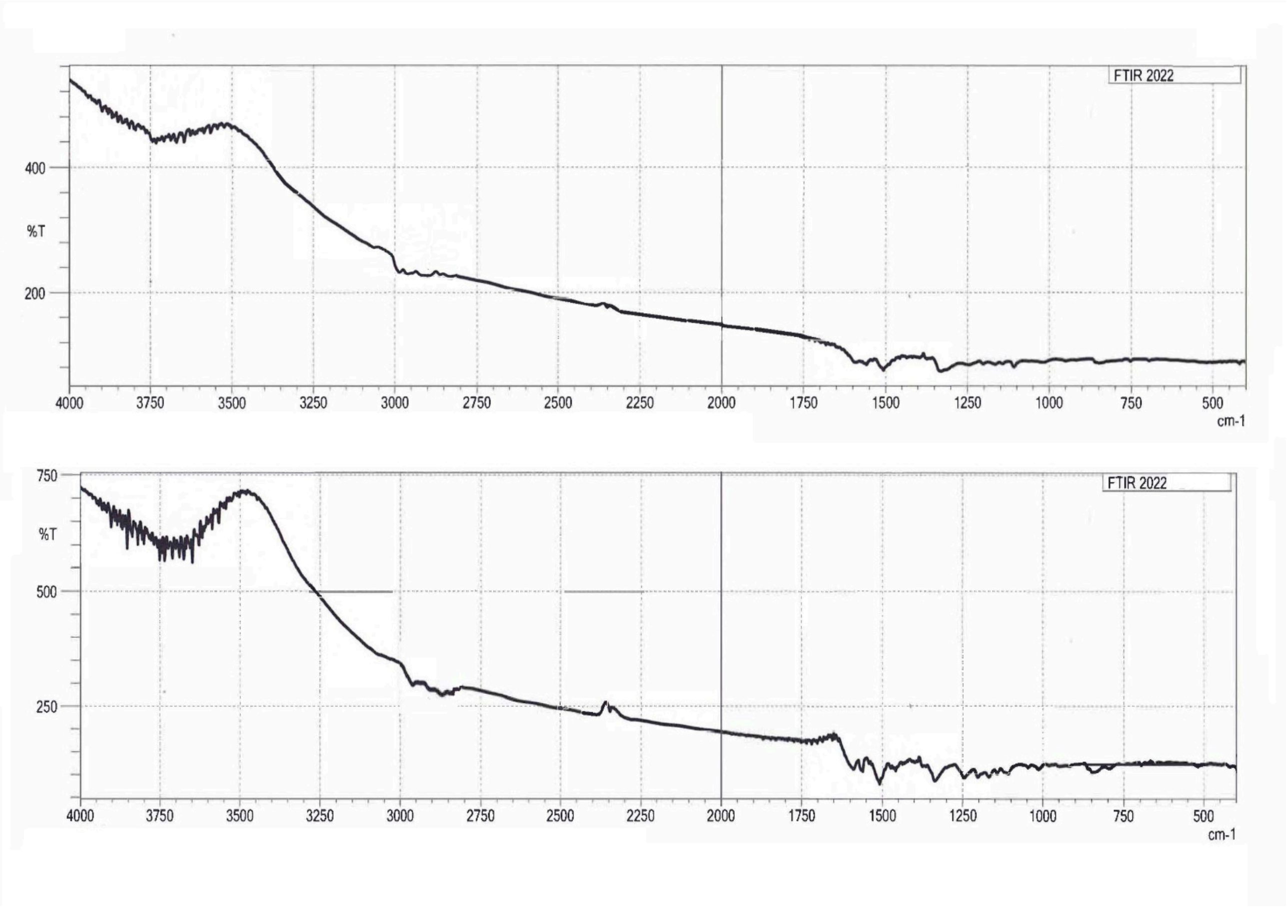

Figure S4. FT-IR spectra of of L1 (above) and Pd1(below) in KBr

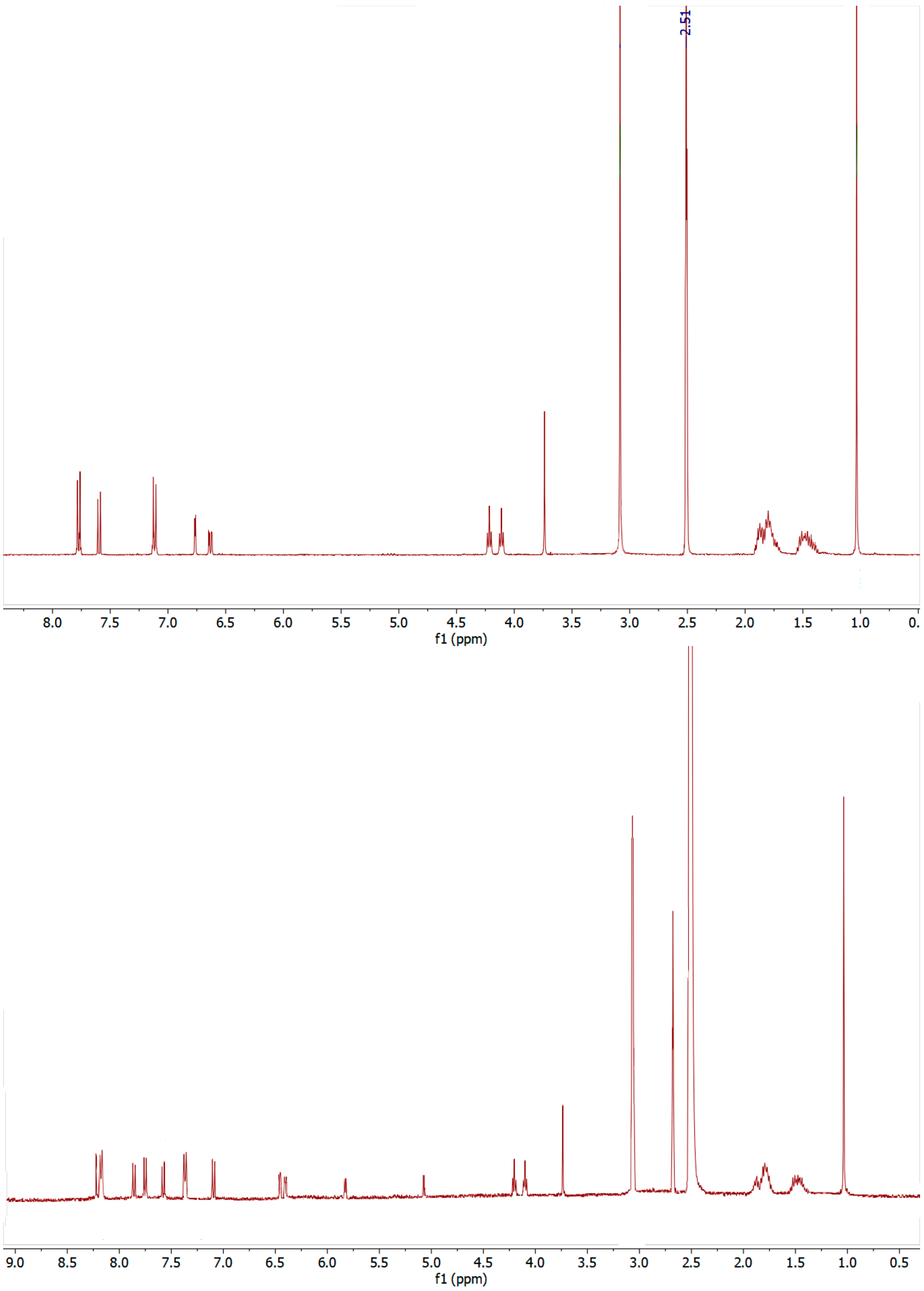

Figure S5. ^{1}H-NMR (400 MHz, DMSO-d6, 25 °C, ppm) spectra of L2 (above) and Pd2(below)

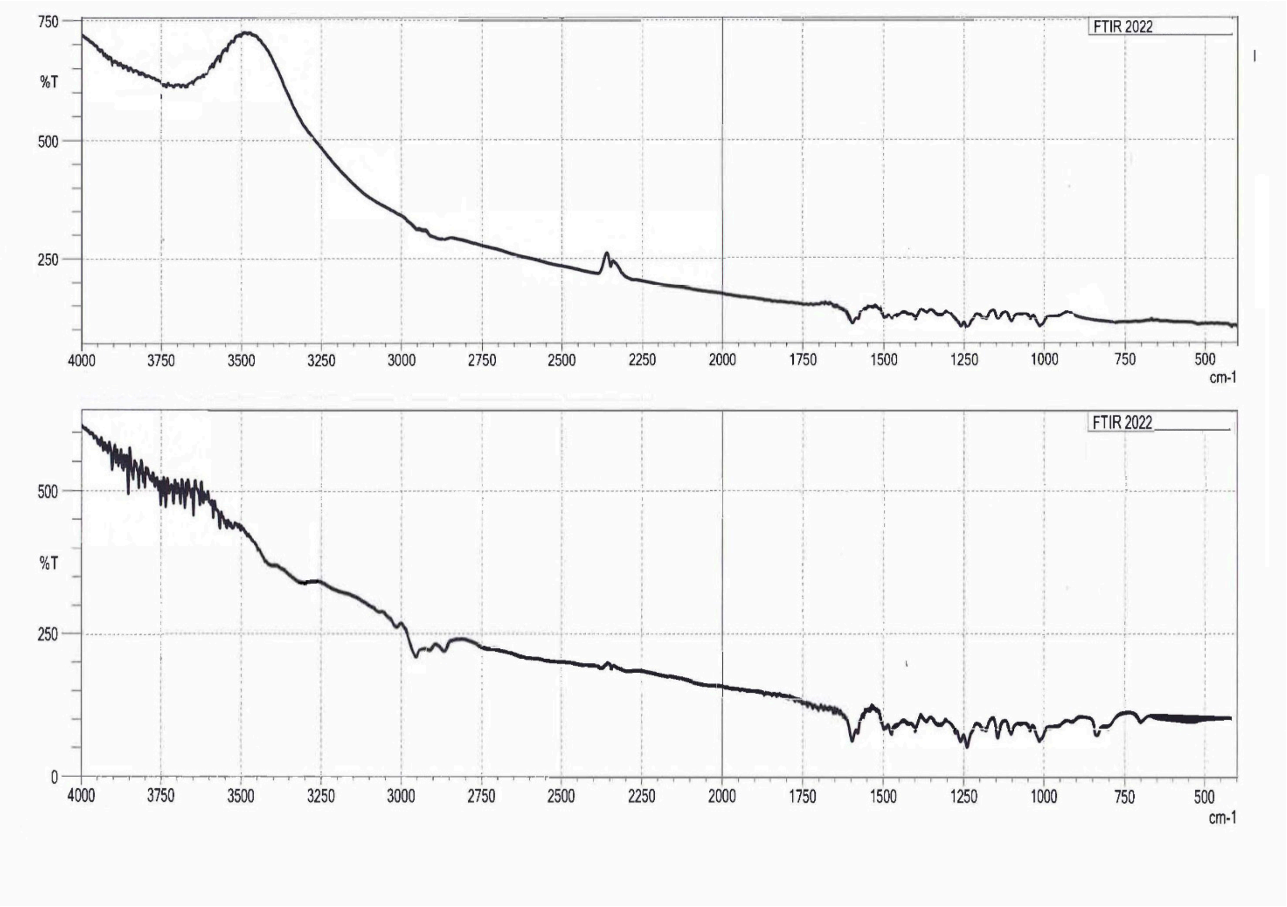

Figure S6. FT-IR spectra of of L2 (above) and Pd2 (below) in KBr
